# Role of Vitamin K in Bone and Muscle Metabolism

**DOI:** 10.1007/s00223-022-00955-3

**Published:** 2022-02-12

**Authors:** N. Alonso, A. Meinitzer, E. Fritz-Petrin, D. Enko, M. Herrmann

**Affiliations:** Clinical Institute of Medical and Chemical Laboratory Diagnostics, Medical University of Graz, Graz, Austria

**Keywords:** Vitamin K, Vitamin K metabolites, Osteoporosis, Sarcopenia, Mass spectrometry

## Abstract

Vitamin K, a cofactor for the γ-glutamyl carboxylase enzyme, is required for the post-translational activation of osteocalcin and matrix Gla protein, which play a key role in bone and muscle homeostasis. In vivo and in vitro models for osteoporosis and sarcopenia suggest the vitamin K could exert a positive effect in both conditions. In bone, it increases osteoblastogenesis, whilst decreases osteoclast formation and function. In muscle, it is associated with increased satellite cell proliferation and migration and might play a role in energy metabolism. Observational trials suggest that high levels of vitamin K are associated with increased bone mineral density and reduced fracture risk. However, interventional studies for vitamin K supplementation yielded conflicting results. Clinical trials in sarcopenia suggest that vitamin K supplementation could improve muscle mass and function. One of the main limitations on the vitamin K studies are the technical challenges to measure its levels in serum. Thus, they are obtained from indirect sources like food questionnaires, or levels of undercarboxylated proteins, which can be affected by other environmental or biological processes. Although current research appoints to a beneficial effect of vitamin K in bone and muscle, further studies overcoming the current limitations are required in order to incorporate this supplementation in the clinical management of patients with osteosarcopenia.

## Introduction

Osteoporosis is a complex disease characterized by low bone mineral density and alterations in the bone quality, which leads to the appearance of fractures. Bone mineral density is assessed by Dual-energy X-ray absorptiometry (DXA) scan and patients with osteoporosis present T-score values below − 2.5 standard deviations from the average value of young healthy women. Bone quality is defined as the combination of bone composition and structure that contributes to bone strength [1]. Osteoporosis is associated with an increased morbidity and mortality and causes a major public health burden. It commonly appears after the 5th decade of life and can be also associated with loss of muscle function and mass, known as sarcopenia [2]. To date, there are different treatments for osteoporosis which range from dietary supplements like vitamin D and calcium to pharmacological approaches, including antiresorptive (i.e. bisphosphonates) and anabolic drugs (i.e. teriparatide). Some countries, like Japan, have also included vitamin K in their recommendations for patients with osteoporosis [3]. Vitamin K is a liposoluble compound with a well-established function in blood clotting [4], which has also shown some beneficial effects in bone and muscle metabolism, especially in in vitro and in vivo models. Observational and interventional studies in patients with osteoporosis and sarcopenia have yielded conflicting results. This review aims to summarize the latest findings on the role of vitamin K in bone and muscle metabolism, including both preclinical and clinical studies published from 2015.

## Chemical and Physiological Characteristics of Vitamin K

Vitamin K (VK) is an essential cofactor for the γ-glutamyl carboxylase (GGCX) enzyme that converts glutamic acid (Glu) residues into γ-carboxyglutamic acid (Gla) in the Gla protein family [5], which includes seventeen members, namely osteocalcin (OCN), matrix Gla protein (MGP), growth arrest specific protein 6 (Gas6), periostin, periostin-like factor, Gla-rich proteins, seven proteins involved in blood clotting, two proline-rich Gla proteins (PRGP1, PRGP2) and two transmembrane Gla proteins (TMG3, TMG4) [6]. GGCX is located in the endoplasmatic reticulum [5] and expressed in different tissues, including liver, brain, heart, kidney, lung, pancreas and skeletal muscle [7].

**Fig. 1:**
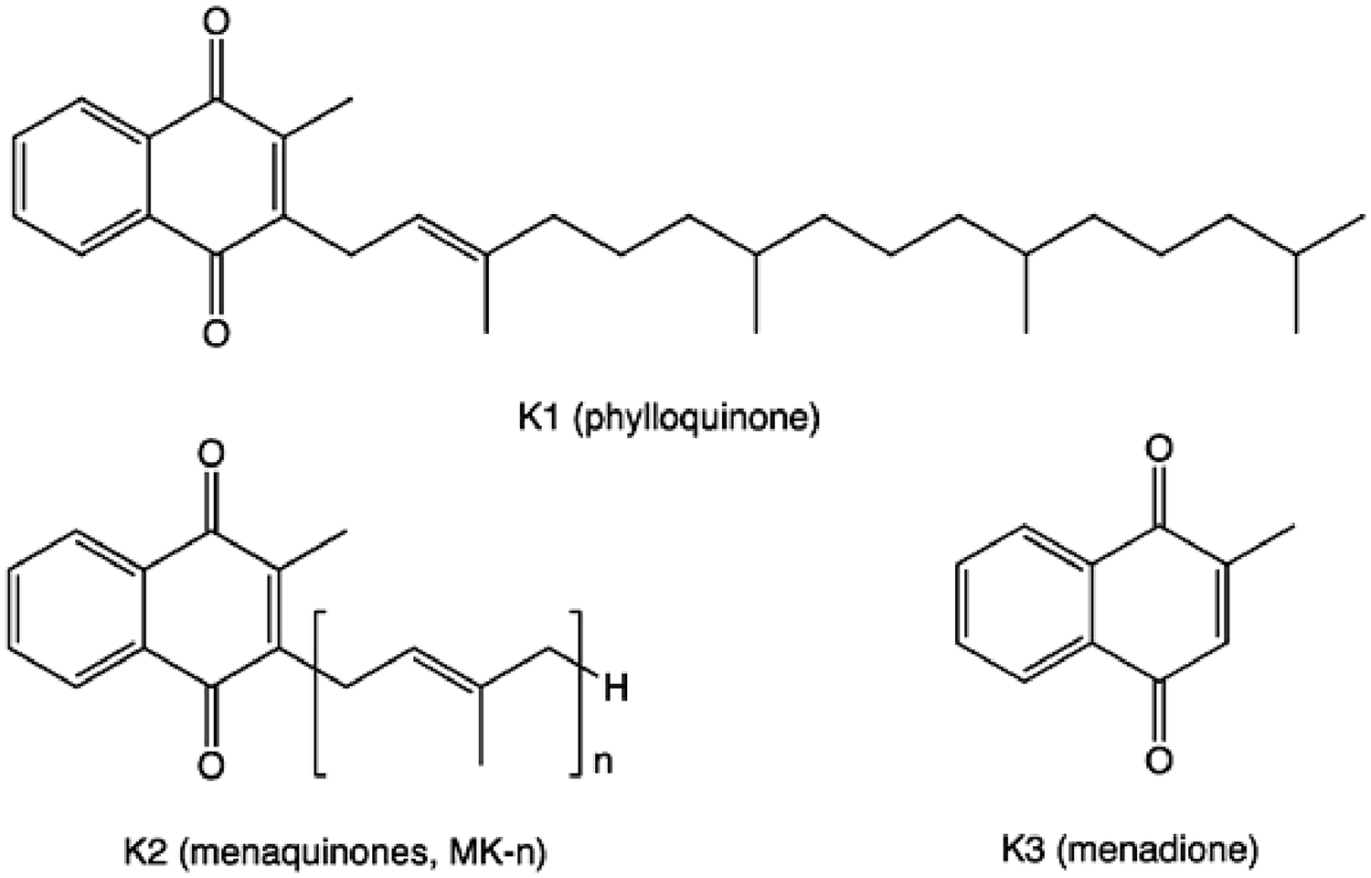
Chemical structure of vitamin K species. The isoprenyl unit is shown in brackets

The term vitamin K comprises a heterogeneous group of chemical compounds of different origin. All of them share an unsubstituted naphthoquinone core structure with a free methyl group in position 2. Different lipophilic side chains in position 3 characterize the individual members of the VK family. These side chains vary in length and the degree of saturation, and thus determine the fat solubility of each compound [8]. Vitamin K-1 (VK-1) and vitamin K-2 (VK-2) are the two main forms of VK that differ only in their side chain in position 3. VK-1 has a phytyl side chain, whereas VK-2 has a side chain with a varying number of isoprenyl units (Fig. 1), an unsaturated hydrocarbon motif with the formula CH2 = C(CH3) − CH = CH2. VK-1, known as phylloquinone, is mostly found in green plants, like spinach, broccoli, green leaf lettuces or parsley, and accounts for more than 50% of the dietary intake of VK [9]. VK-2 (menaquinone) is found in fermented soybeans (natto), egg yolk and chicken breast, and it can also be produced by the gut bacteria. Several forms of VK-2 are known, usually abbreviated as MK-n, where ‘n’ is the number of isoprenyl groups in the side chain. These include MK-1, MK-4, MK-7, MK-9 and MK-11, whereby the properties and activities depend on the lipophilic side chain and the aromatic methyl-group ring. Vitamin K-3 (VK-3), also known as menadione (2-methyl-1,4-naphtoquinone) is a water-soluble synthetic form of VK which has no side chain and is classified as a pro-vitamin [10]. Small amounts of VK-1 can be converted into MK-4 either directly within certain tissues or via the VK-3 intermediate which is prenylated to be converted into MK-4 by the ubiquitously expressed UBIAD1 enzyme [11].

**Fig. 2:**
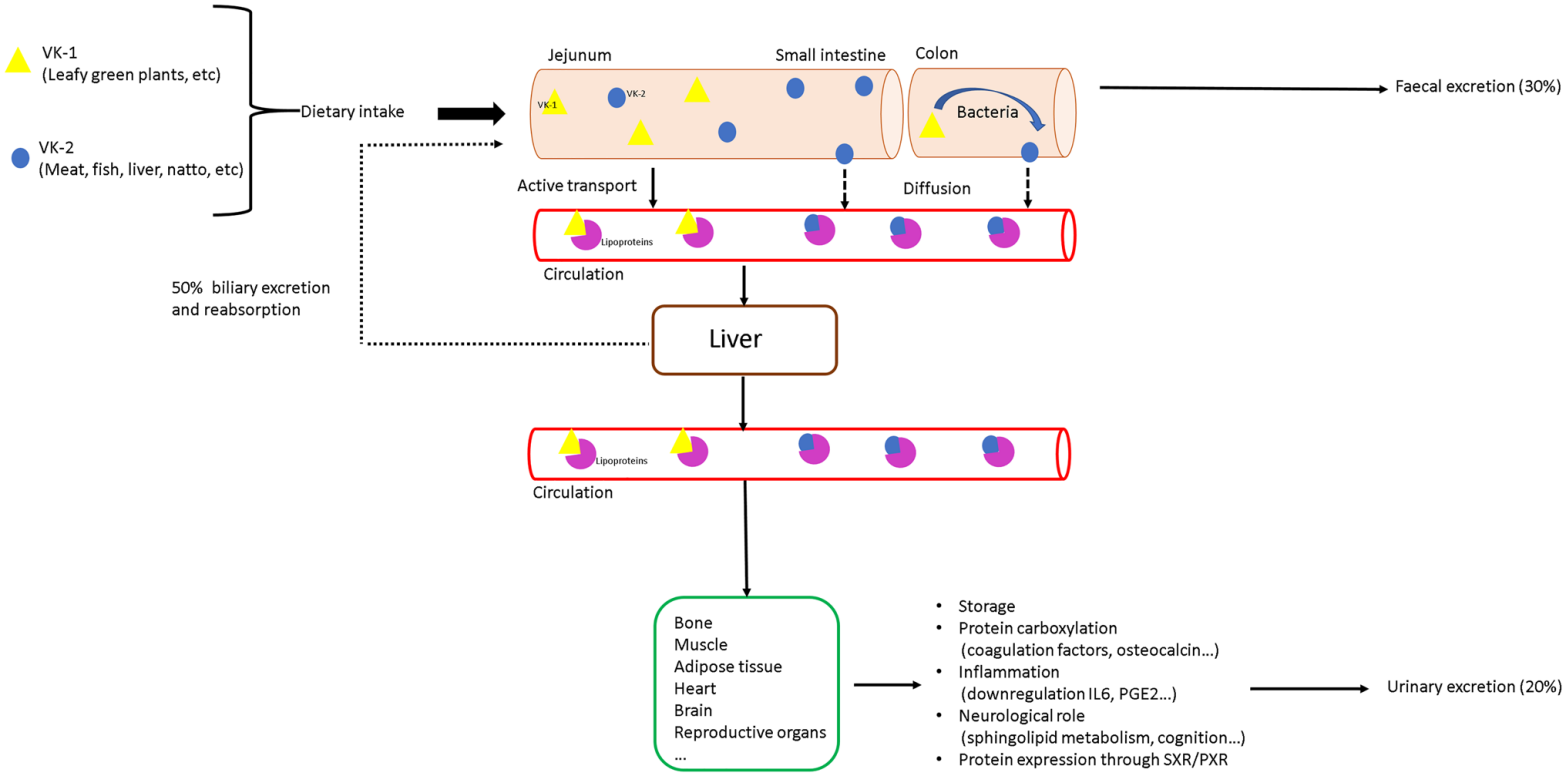
Vitamin K uptake and elimination in the body: After oral intake of vitamin K, it is absorbed in the intestine into the thoracic duct and then transported by the bloodstream to the liver (where it undergoes further metabolism and participates in various carboxylation reactions of the blood coagulation factors), adipose tissue (probably for storage), muscles and bones. Approximately 50% of the excretion is reabsorbed, 30% is eliminated via the faeces and approximately 20% via the urine

Dietary VK-1 is absorbed in the intestine by active transport in the jejunum, whilst VK-2 is absorbed in the small intestine by diffusion. Bile salts and products of pancreatic lipase are required for optimal absorption. Therefore, extrahepatic cholestasis and pancreatic insufficiency may result in VK deficiency [12]. The absorption rates of VK metabolites decrease significantly with the length of the side chain [13]. In the blood circulation, VK is transported to the different tissues by lipoproteins (Fig. 2). Bioavailability and pharmacokinetics vary widely amongst the different forms of VK. Some of them have very short half-life, like VK-1 [14], and can be found in the circulation from 4 to 10 h after ingestion, whilst others, like MK-7, are present for more than 96 h [15]. Tissue distribution also differs between the short-chain and long-chain VKs. VK-1 can be found in all tissues, but mostly in liver and heart. MK-4, instead, is most abundant in pancreas, salivary gland and sternum [16].

**Fig. 3:**
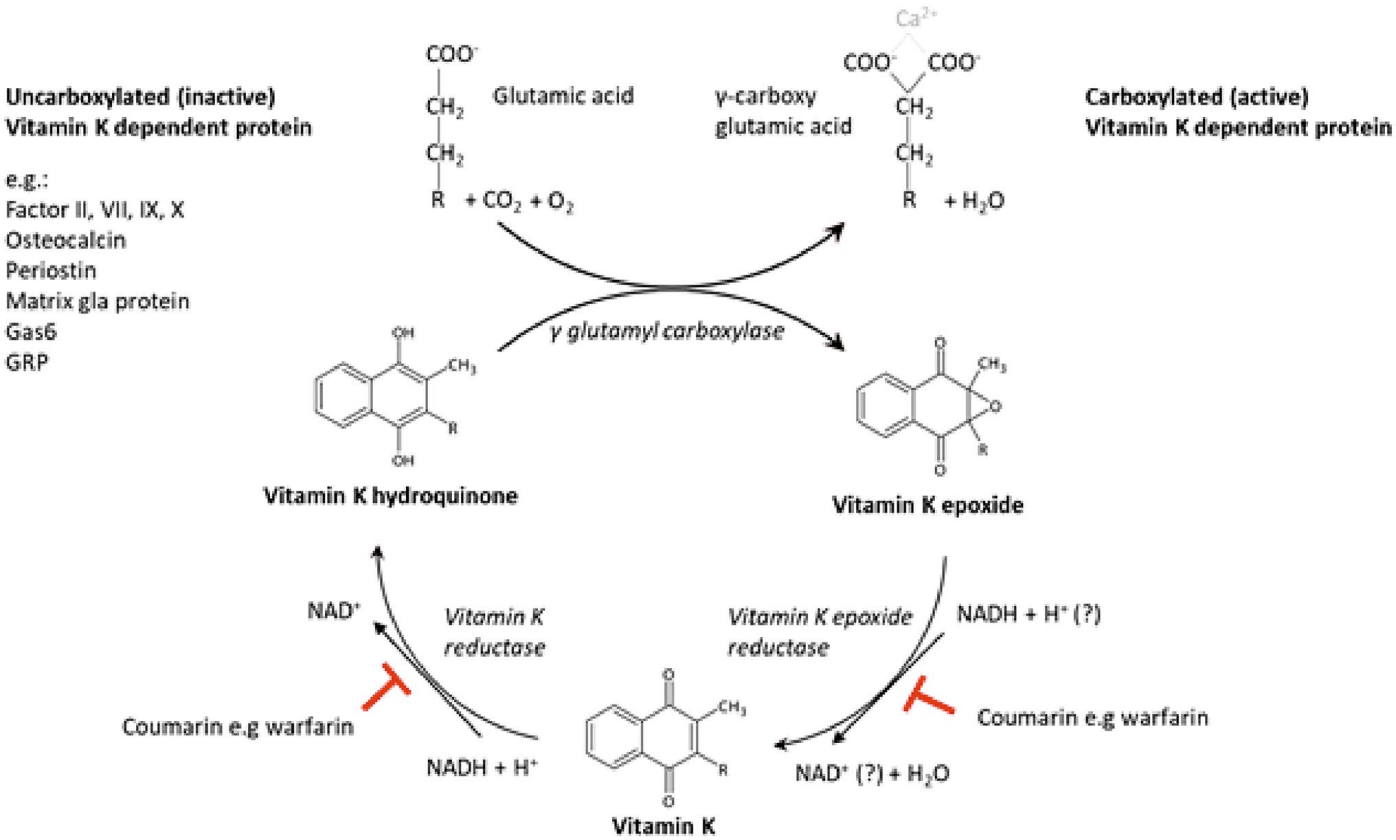
Schematic representation of the vitamin K cycle

Once the VK is incorporated into the rough endoplasmic reticulum of the cells, it hydroxylates into the biologically active form hydroquinone. This form acts as a cofactor of GGCX and is converted into VK-2,3 epoxide, then reduced into the respective quinone by VK epoxide reductase complex subunit 1 (VKORC1), and, finally, it is transformed back into hydroquinone by the VK reductase. Then, the cycle starts again. Various drugs, such as coumarin (i.e. warfarin), inhibit the VK epoxide reductase (Fig. 3) [17].

The daily recommended intake of VK it is not well established yet and varies from country to country. It ranges from 90 µg (women) and 120 µg (men)/day in the USA to 70 ug/day in all adults including pregnant and lactating women in Europe (European Food Safety Authority, EFSA 2017) [9, 18]. VK-1 accounts for 50%, MK-4 for 10% and MK-7, MK-8 and MK-9 for 40% of the absorbed VK [19]. However, it is unclear whether these recommendations can be sufficient for the total of all carboxylation reactions. Recent studies have shown that only MK-7 can achieve the γ-carboxylation of the extrahepatic vitamin K-dependent proteins (VKDPs) with the recommended daily requirement [20].

Although diet is the major source of vitamin K, some VK metabolites are produced by the gut microbiota. *Bacteroides fragilis* produces MK-10, MK-11 and MK-12, *Eubacterium lentum* generates MK-6 and *Lactococcus lactis ssp.lactis* and *ssp.cremoris* synthesize MK-8 and MK-9 [21, 22]. Moreover, most aerobic Gram-positive and anaerobic gut bacteria use VK-2 in their electron transport pathways and are also responsible for the length and saturation of the VK side chain [23]. However, the exact mechanism is not fully understood yet.

## Analytical Methods of Vitamin K Metabolites

VK metabolites are present in serum at very low concentrations, which limits their quantification with the methodology available to date. Besides, they show high affinity for lipoproteins, making their separation technically challenging. Therefore, most of the studies on VK assess its levels by food questionnaires or other proxy parameters, like prothrombin rate or levels of undercarboxylated proteins.

The first determination of VK-1 in serum was reported in 1979 by Lefevere et al. [24] using a classical chromatographic method with direct ultraviolet detection that required large sample volumes and a time-consuming pre-analytical procedure. This method suffered from a limited sensitivity and selectivity. A few years later, Ueno et al. developed a first HPLC method with electrochemical detection that offered improved sensitivity and specificity [25]. Through the employment of a glassy carbon electrode and an Ag/AgCl reference electrode, they were able to detect 2.2 nmol/L of VK-1 in serum. The next significant improvement in the analysis of VK was the introduction of a post-column reduction reactor filled with metallic zinc coupled to a fluorescence detector [26]. This widely used approach results in an enhanced sensitivity and selectivity. The improvement in separation columns over the last 20 years allowed the use of less complex sample preparation procedures for the removal of interfering substances. Today, the HPLC approach with post-column reduction reactor and fluorescence detector is the most commonly used method for the measurement of VK in medical laboratories. In the absence of a standard reference material and an accepted reference measurement procedure, the comparability of results within a single laboratory as well as between laboratories is limited and depends on the preparation of the post-column reactor, the mobile phases and the choice of separation column.

Liquid chromatography-tandem mass spectrometry (LC–MS/MS) has brought significant progress in analytical chemistry through its high selectivity and sensitivity. However, its use for the measurement of VK is hampered by the non-polar structure of VK and the high number of accompanying substances that suppress ionization and thus limit sensitivity. In order to optimize sensitivity, LC–MS/MS methods usually employ the same sample preparation as HPLC, which consists of liquid–liquid extraction followed by a solid-phase purification step [27]. In addition to this rather complex sample preparation, Suhara et al. used a 75-min chromatographic separation protocol with extensive wash and re-equilibration steps. Compared to previous methods, this approach resulted in improved sensitivity and selectivity. With 500 µL of sample volume, they achieved a limit of detection (LOD) of 0.09 nmol/L for VK-1, 0.11 nmol/L for MK-4 and 0.12 nmol/mL for MK-7. Furthermore, the serum concentrations for VK-1, MK-4 and MK-7 correlated well with the HPLC method. When employing this LCMS method in a cohort study of 396 Japanese women, Tsugawa et al. found mean plasma concentrations of VK-1, MK-4 and MK-7 that ranged from 0.88 for MK-4 to 14.3 nmol/L for MK-7 [28]. Despite its improved sensitivity and selectivity, this rather complex method is not suitable for clinical practice.

In 2014, Karl et al. [29] developed a method for the simultaneous quantitation of 11 VK species (VK-1, MK-4 to MK-13) by LC–MS/MS with atmospheric pressure chemical ionization (APCI). However, the advantage of detecting multiple VK species in one run is flawed by a limited sensitivity (LODs between 1 and 30 nmol/L). Consequently, most VK compounds could only be quantitated in stool, but not in serum.

Riphagen et al. have tried to develop a less complex, but sufficiently sensitive, method for the measurement of VK by focussing on the most abundant species VK-1, MK-4 and MK-7 [30]. The limits of quantification (LOQ) using 350 µL plasma ranged from 0.14 nmol/L for VK-1 and MK-4 to 4.4 nmol for MK-7. In 2019, Dunovska et al. [31] published a method with an improved sensitivity for these three compounds with LOQs of 0.07 nmol/L for VK-1 and MK-4, and 0.05 nmol for MK-7.

In summary, the measurement of VK is highly complex and requires a compromise between sensitivity and throughput (Table 1). Amongst all methods published to date, the HPLC method from Haaron et al. and the LC–MS/MS method from Dunovska et al. are best suited for clinical practice. The lack of standardization and appropriate external quality assurance programmes limit the comparability of results obtained by different laboratories. So far, the European Vitamin K External Quality Assurance (KEQAS) programme is the only one that allows an independent control of VK-1 results, but does not provide a certified target concentration. Other VK species, such as MK-4, MK-7 and vitamin K1 2,3-epoxide are only available as a pilot scheme with concentrations outside the physiological range. Therefore, some laboratories consider assessing the levels of vitamin K using indirect methods, like measuring prothrombin time (PT) or undercarboxylated vitamin K-dependent proteins. Vitamin K deficiency in adults is clinically characterized by a bleeding tendency due to the low activity of blood coagulation factors, resulting in an increase of prothrombin time or partial thromboplastin time. In healthy adults, this can occur more than 2–3 weeks after the very low phylloquinone intake (i.e. < 10 lb/day) [32]. However, this measure is not a reliable indicator of vitamin K status, because an abnormal prothrombin time can also arise from hepatic dysfunction or haematological conditions, independently of vitamin K (reviewed by Booth and Al Rajabi [33]). Alternatively, the determination of undercarboxylated vitamin K-dependent proteins is also used as a proxy to identify vitamin K deficiency. Serum concentration of undercarboxylated osteocalcin (ucOC) [34, 35] or desphospho-uncarboxylated MGP (dp-ucMGP) are two examples. These undercarboxylated proteins can be determine alone, or together with the total amount of each protein expressing the results as percentage. Although the direct quantification of dp-ucMGP is capable of showing a reduction in response to vitamin K supplementation [36–38], expressing dp-ucMGP as percentage of the total MGP concentration is more precise because it takes into consideration the biological variability of the total MGP concentration. Moreover, the PIVKA-II (Protein Induced by Vitamin K Absence II) concentration has also been proposed as a biomarker for vitamin K status in some renal, bone and cardiovascular diseases [39–41]. Although the Expert Group of the EFSA [18] acknowledges that phylloquinone intake at certain doses changes the concentration of the abovementioned proteins, it has to be mentioned that these doses are considerably higher than those recommended in Europe. Another issue is that commercially available PIVKA-II assays are not sufficiently sensitive to measure normal concentrations, which are ≤ 2 µg/L. Also, the optimal degree of carboxylation of PIVKA-II, OC or MGP is unknown and reliable information on vitamin K intake required for c-carboxylation of these proteins is lacking. In summary, the inherent limitations of indirect vitamin K surrogate markers make them rather unreliable and thus they should be used with caution. The direct measurement of vitamin K and its metabolites can overcome virtually all of these issues and is thus the preferred way to assess vitamin K status.

**Table 1:** Overview of the development of the vitamin K determination in human serum and the technologies used

Study, year [ref]	Volume	Instrumentation/detection	Sample preparation	LOQ (nmol/L)	Fasting human serum levels (nmol/L)
Lefevere et al., 1979 [24]	2 mL	HPLC / UV 248 nm	Methanol/n-hexan & precleaning on a silica column	1.1 nmol/L VK-1	11 to 66 nmol/L
Ueno et al., 1983 [25]	1.5 mL	HPLC / ECD	Ethanol/n-hexan & precleaning on a silica column	0.7 nmol/L VK-1	2.2 nmol/L (n = 26)
Haroon et al., 1986 [26]	0.5 – 1 mL	HPLC / FLD Zn post-column reactor	Ethanol/n-hexan & solid-phase extraction (SPE)	0.1 nmol/L VK-1	1.23 nmol/L (*n* = 22)
Suhara et al., 2005 [27]	1 mL	LC-APCI-MS/MS	Ethanol/n-hexan & solid-phase extraction (SPE)	0.09 VK-1; 0.11 MK-4, 0.12 MK-7	2.68 ± 1.25 VK-1; 0.88 ± 1.04 MK-4; 4.3 ± 11.5 MK-7 (*n* = 20)
Riphagen et al., 2016 [30]	0.35 mL	LC-APCI-MS/MS	Precipitation with ethanol & solid-phase extraction (SPE)	0.14 VK-1; 0.14 MK-4, 4.4 MK-7	1.35 (0.89–2.32) VK-1,; 0.20 (0.17–0.25) MK-4; < 4.40 MK-7 (*n* = 60)
Klapkova et al., 2018 [101]	0.5 – 1 mL	HPLC / FLD Zn post-column reactor	Ethanol/n-hexan & solid-phase extraction (SPE)	0.07 VK-1; 0.07 MK-4; 0.05 MK-7	1.08 ± 0.88 VK-1; 1.85 ± 0.60 MK-4; 1.83 ± 1.66 MK-7 (*n* = 158)
Dunovska et al., 2019 [31]	0.5 mL	LC–ESI–MS/MS	Ethanol/n-hexan & solid-phase extraction (SPE)	0.07 VK-1; 0.07 MK-4; 0.05 MK-7	0.44 (0.07–4.8) VK-1; 0.42 (0.11–6.3) MK-4; 0.14 (0.08–2.1) MK-7 (*n* = 191)

## Pre-clinical Studies on the Role of Vitamin K in the Musculoskeletal System

### Vitamin K and Bone

**Fig. 4:**
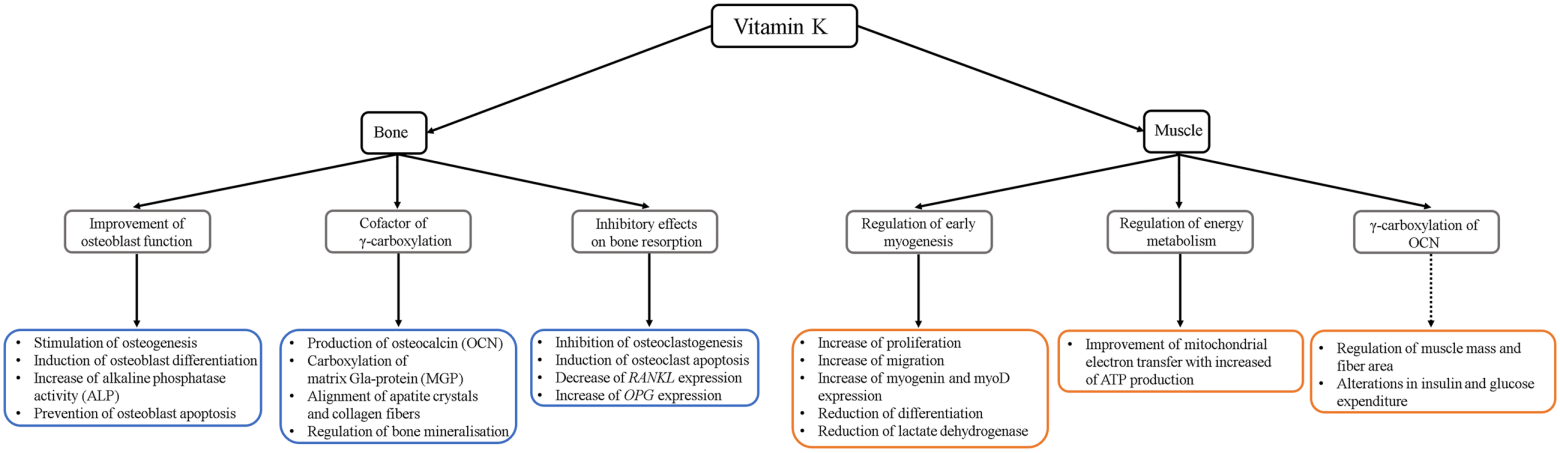
Role of vitamin K in bone and muscle metabolism from in vitro and in vivo studies. Dashed line indicates a questionable role

Preclinical research has provided essential insights into the metabolic activities of VK in the musculoskeletal systems. Cell culture studies indicate an anabolic role of VK in bone by increasing osteoblast activity and decreasing osteoclastogenesis. Already more than 20 years ago, in vitro studies found that VK-2 promotes mesenchymal stem cell proliferation and differentiation into osteoblasts, but not adipocytes [42], by enhancing the expression of cartilage-associated Growth differentiation factor 15 (Gdf15) and Stanniocalcin 2 (Stc2) [43]. It also prevents osteoblast apoptosis by inhibiting the apoptotic genes *Fas* and *Bax* [42] and stimulates autophagy via Lc3 and Beclin1 [44]. A very recent study has shown that MK-7 is required for osteogenic differentiation at early stages, when it upregulates RUNX2 through an OCN-independent pathway [45] (Fig. 4). Apart from enhancing osteoblast differentiation and activity, VK also regulates osteoclasts, both directly and indirectly. It inhibits osteoclastogenesis by IκBα-dependent NF-κB downregulation [46] and induces osteoclast apoptosis [47], as well as reduces the *RANKL/OPG* mRNA ratio in osteocytes, leading to a decrease in osteoclast activity [48] (Fig. 4).

In differentiated osteoblasts, VK acts primarily as a cofactor for GCCX, which activates bone-related proteins, like osteocalcin (OCN) and matrix Gla protein (MGP). OCN is the most abundant non-collagenous protein in the bone matrix. It is synthesized by osteoblasts and released to bind hydroxyapatite crystals in the extracellular matrix, promoting mineralization. Its affinity for calcium depends on the carboxylation of three Glu residues by the two enzymes GGCX and VKORC1 [49]. Most of the carboxylated OCN is bound in the extracellular bone matrix, but during bone resorption, the low pH in the resorption lacunae can decarboxlyte Gla residues of OCN and resulting in the release of OCN, and, in particular, undercarboxylated OCN, into the blood. Therefore, the OCN concentration in serum is used as a biomarker for bone turnover, and undercarboxylated OCN has been proposed as an indicator of the VK levels [50]. Recent results from an OCN knockout mouse model suggest that OCN is required for aligning the hydroxyapatite crystals between collagen fibres and thus contributes to bone quality (Fig. 4) [51]. However, the importance of the VK-dependent OCN carboxylation for bone mineralization is questioned by the results of another conditional knockout (KO) model for *GGCX* (*Col1-Cre Ggcx*^−/−^) [52]. In this model, the lack of GGCX in the osteoblastic lineage resulted in increased trabecular and cortical bone formation and extra skeletal calcification, but no reduction in bone mineral density.

In addition to OCN, VK is also required to carboxylate MGP, expressed in cartilage, bone and soft tissue. This protein binds the organic matrix and hydroxyapatite crystals and inhibits vascular calcification [53]. Apart of its role in inhibiting bone formation, it also regulates osteoclastogenesis. MGP inhibition leads to an increased osteoclast differentiation and bone resorption as demonstrated in vivo in a *Mgp*^*−/−*^ mouse model [54]. Moreover, VK is needed for the carboxylation of the growth arrest specific gene product (Gas6), which regulates the activity of osteoclasts and protein S, a cofactor for the anticoagulant protein C. It shows a high degree of homology with Gas6, but its role in bone is still unknown [55].

Through binding to the steroid and xenobiotic receptor (SXR; also known as pregnane X receptor, Pxr, in mice), VK also exerts effects that are unrelated to γ-carboxylation [56]. SXR is a xenobiotic sensor associated with detoxification and drug excretion that activates Tsukushi, Matrilin-2 and CD14 proteins, involved in collagen accumulation, osteoblast- and osteoclastogenesis (Fig. 4) [5]. Several Pxr KO mouse models have been developed, showing different phenotypes. One of them presented hypophosphatemia, as Pxr directly activates the cotransporter Na/Pi Slc34a2 [57], whilst the other did not show any change in serum calcium or phosphate, but an osteoporotic phenotype: reduced bone mineral density (BMD) and decreased trabecular and cortical thickness with increased bone fragility [58].

Last, but not least, there is also some evidence that VK-1 and VK-2 are involved in the antioxidant defence against reactive oxygen species that are produced by metabolically active osteoblasts [45, 59]. MK-7 also increases the expression of PMCA, a calcium transporting ATPase, promoting normal bone mineralization even under oxidative stress conditions [59].

In addition to the isolated effects of VK in bone, several in vitro and in vivo studies have investigated if VK modifies the effects of common anti-osteoporotic treatments. For example, combining vitamin D3 and vitamin K has resulted in additive anabolic effects in murine models and osteopenic patients. In a murine model for diabetes, the two vitamins together increased calcium deposits, bone anabolic markers and bone formation transcription factors, suggesting that this combination could be useful for the treatment of diabetes-associated osteoporosis [60]. In cultured precursor cells from osteopenic patients, the combined treatment with VK and vitamin D increased osteoblastogenesis and MSC differentiation into osteoblasts and decreased osteoclastogenesis. After one year of treatment, these patients presented an increased lumbar spine and femoral neck BMD and a decreased risk of fracture [61].

Genistein, an estrogenic agent, also showed an increased bone formation capacity when combined with vitamin K. Both treatments increased OCN and alkaline phosphatase activity in MC3T3 cells, suggesting that this treatment could promote osteoblast activity [62].

Teriparatide is a well-established anabolic treatment for patients with severe osteoporosis. The combined treatment of ovariectomized rats, an established model for osteoporosis, with teriparatide and VK-2 increased serum γ-carboxylated OCN, showed higher BMD [63] and femoral bone strength [64] than each of the two treatments by itself. In vitro analysis of primary bone marrow-derived stromal cells from these animals showed that the combined treatment prevented bone loss and enhanced bone formation and mineralization better that teriparatide alone [65].

Moreover, vitamin K also seems to have beneficial effects when combined with zoledronic acid, an antiresorptive drug for osteoporosis. VK-2 pre-treatment of primary murine osteoblasts reduces Sost expression and partially prevents the inhibition of bone formation indices (BMD, calcium content and bone strength) caused by zoledronic acid [66]. In vivo studies using ovariectomized Wistar rats confirmed these results, as pre-treatment with VK increased the calcium content, improved trabecular structure and anti-pressure strength of bone after bisphosphonate treatment. However, no effect was detected when the treatments were administered at the same time [66].

Taken together, existing evidence from preclinical studies is limited and in many ways inconclusive. However, there seem to be some anabolic effects that are mediated by stimulating osteoblasts and inhibiting osteoclasts. In addition, VK seems to improve the efficacy of common bone-preserving compounds, such as vitamin D, teriparatide or bisphosphonates. The underlying mechanisms are poorly understood and subject of ongoing research.

### Vitamin K and Skeletal Muscle

Little is known about the role of VK supplementation in the skeletal muscle. Only few studies have been performed to date, suggesting that VK exerts a protective effect. Most of the research, however, has focussed on how the carboxylation of bone-derived OCN could affect muscle health, a role that lately has been questioned [51, 67]. In vitro studies by Rønning et al. have shown that MK-4 increases proliferation and migration of bovine muscle cells [68]. Furthermore, MK increases the expression of myogenin and myoD, and reduces the differentiation of bovine satellite cells. In this cell culture model, MK-4 also reduces lactate dehydrogenase, a marker for cell lysis and toxicity, indicating a stabilizing effect on muscle cells. Expression of Lrp1 and Ldlr receptor was increased shortly after the treatment with MK-4, and the authors suggest that they could be involved in the VK-driven changes in muscle cells, although the exact mechanism is still unknown (Fig. 4).

Some studies also suggested that VK could regulate energy metabolism in the skeletal muscle. VK-2 (MK-4) improves the transfer of electrons in the mitochondria, increasing ATP production [69]. It could as well maintain the balance of the skeletal muscle mitochondria, which is a major factor to prevent sarcopenia [70].

Moreover, for long time OCN has been considered a bone hormone that could regulate different tissues, including muscle [71]. This work has been based on a knockout mouse model for OCN [72], which showed reduced muscle mass and decreased muscle fibre area compared to wild type animals. These animals also presented alterations in insulin secretion and glucose expenditure by peripheral tissues, like liver, muscle and adipose tissue (reviewed in [73]). However, last year, two independent OCN KO mouse models failed to validate these changes [51, 67]. The animals exhibited normal muscle mass and fibre area, and no alterations in glucose or lipid metabolism, which questions the hormonal role of OCN.

## Role of Vitamin K in Human Studies

### Vitamin K and Fragility Fractures

Preclinical analyses suggest that VK is involved in bone metabolism and has anabolic effects. However, it is unclear if VK has an influence on bone metabolism and bone quality in humans. Over the years, a number of studies has been published with conflicting results. Several recent observational studies suggest a relationship between a high VK intake and increased BMD (Table 2), in both East Asian and European populations. However, most of these studies are limited by a rather small cohort size and the lack of VK measurements in serum or plasma. In addition, the study populations were rather heterogeneous including healthy elderly individuals, osteoporotic women, cystic fibrosis patients, renal patients and children with low trauma fractures. Apart from three studies [74–76], VK status was estimated indirectly by food questionnaires or serum levels of undercarboxylated proteins (OCN or MGP). When measuring serum VK by HPLC or LC–MS/MS, no relationship with BMD or biochemical bone turnover markers was found [74, 75].

**Table 2:** Observational studies for the effect of vitamin K in bone mineral density and fractures since 2015

Study, year [ref]	Population	N	ucOCN	Serum VK	VK measure method	BMD	BTM	Fracture risk	Other
Kim et al., 2015 [102]	Korean adults	7092	–	–	–	Decrease in women with low VK intake	–	–	–
Popko et al., 2018 [103]	Caucasian children with low energy fractures	39	yes	–	–	–	No differences (NTx, BALP, Calcium, vitD)	Decreased with better VK status	–
Evenepoel et al., 2019 [104]	Adult patients (mostly Caucasian) with CKD	468	–	–	–	Decrease when high ucMGP	No change	Increased when low ucMGP	–
Nalevaiko et al., 2021 [105]	Men and women on anticoagulants (DOACs or warfarin) for at least 1 year	150	–	–	–	Decrease (more with warfarin)	–	–	Decreased TBS (more with warfarin)
Finnes et al., 2016 [76]	Caucasian men and women 65–79 years old	2331	–	Yes	HPLC	–	–	Decreased hip fx in high VK1 and vit D	–
Tejero et al., 2016 [75]	Adolescents and adults with cystic fibrosis	50	–	Yes	HPLC	No difference	No difference	–	–
Jaghsi et al., 2018 [106]	Syrian postmenopausal OP women and postmenopausal healthy control women	23	–	Yes	ELISA	Increased with high VK1	–	–	–
Moore et al., 2020 [74]	Postmenopausal women with OP	374	–	Yes	LCMSMS	No difference	–	Increased in low levels of VK1	Increased cross–sectional area, cross sectional moment of inertia and section modulus at the narrow neck of femur; decreased ucMGP in high VK1 levels

*ucOCN* undercarboxylated OCN, *fx* fracture, *TBS* trabecular bone score, *CKD* chronic kidney disease, *LCMSMS* liquid chromatography tandem mass spectrometry

Additional information can be derived from supplementation studies. All 15 VK treatment studies that have been published since 2015 are summarized in Table 3. The results of these studies are inconsistent. Whilst 6 studies reported an increase in BMD upon VK treatment, the others did not. In a meta-analysis of 19 randomized controlled trials, including 6,759 Asian and Caucasian participants, Huang et al. found an increase of vertebral BMD in postmenopausal women with osteoporosis [77]. In non-osteoporotic individuals this effect was not present. Although this meta-analysis suggests that the evidence for VK effect on BMD is robust, it should be mentioned that all osteoporotic patients were of Asian origin, mostly Japanese, whilst the controls were mostly Caucasians. This leaves ample room for ethnicity-related effects, such as differences in pharmacogenomics and lifestyle [77]. In fact, studies in Caucasians are limited to a few rather small studies. The best evidence in Caucasians comes from a randomized placebo-controlled, study involving 142 healthy postmenopausal Danish women [78]. In this study, 12 months of VK-2 supplementation together with calcium and vitamin D did not change BMD. In order to assess whether a longer treatment was needed to induce significant effects, the authors extended their study for another two years [79]. However, even after 3 years of treatment BMD at total hip, femoral neck or lumbar spine was not affected by VK supplementation. Only a small increase in trabecular thickness was observed. The combination of VK-2 and risedronate also failed to improve BMD when compared with risedronate alone in a prospective, multicentre, open-labelled, randomized trial including 1983 patients (Table 3) [80].

**Table 3:** Interventional studies for the effect of vitamin K in bone mineral density and fractures since 2015

Study, year [ref]	Population	N	Duration	Supplementation	ucOCN	Serum VK	VK measure method	BMD	BTM	Fracture risk	Other
Huang et al., 2015 [77]*	Japanese or East Asian, mostly postmenopausal women with OP; Caucasian healthy postmenopausal women	6759	6–48 months	100 ug—45 mg/day MK-4 or MK-7, some with Ca (133.8 mg/day to 2 g/day) or vitamin D3 (0.75-10ug/day). 2 studies received bisphosphonates (17.5 mg/week risedronate or 5 mg/day alendronate)	Yes	–	–	Increased at LS. No changes in non-OP	Decreased ucOCN	Decrease. No changes in non-OP	–
Rønn et al., 2016 [78]	Postmenopausal Caucasian women with osteopenia	142	12 months	375ug MK-7, 800 mg calcium and 38ug vitD, daily	Yes	–	–	No difference	No change in P1NP or CTx; increased BSAP; decreased ucOCN after 3 months	–	Trabecular number did not change in VK but decreased in placebo; trabecular thickness unchanged in VK but increased in placebo
Ebina et al., 2016 [107]	Japanese men and postmenopausal women	160	12 months	50 mg/month minodronate and 45 mg/day VK2 or eldecalcitol 0.75ug/day	–	–	–	Increased	Decreased P1NP	–	–
Shikano et al., 2016 [108]	Japanese patients with glucocorticoid-induced osteoporosis	60	4 weeks	45 mg/day VK2 and 30–60 mg/day prednisolone	Yes	–	–	No difference	Increased P1NP, decreased ucOCN	No difference at 1.5 years	–
Kodama et al., 2017 [109]	Japanese adults with cerebral palsy and osteoporosis	32	12 months	45 mg VK2/day	Yes	–	–	Increased	Decreased ucOCN	–	–
Maria et al., 2017 [61]	Caucasian postmenopausal women with osteopenia	20	12 months	5 mg melatonin, 450 mg strontium (citrate), 2000 IU vitamin D3 and 60ug VK2	–	–	–	Increased	Increased P1NP, reduced CTx:P1NP ratio	reduced for major OP fx	–
Tanaka et al., 2017 [80]	Japanese women over 65 years	1983	2 years	45 mg/day VK2 and 2.5 mg/day or 17.5 mg/week risedronate	Yes	–	–	No difference	Decreased ucOCN at 6 months	No difference	–
Barnuevo et al., 2018 [83]	Healthy Caucasian premenopausal women	181	18 months	Dairy product supplemented with either 1ug of vit D or 1ug vitamin D and 18ug VK2 or nothing else	Yes	–	–	No difference	No difference	–	–
Su et al., 2019 [90] *	Japanese, Chinese and Indonesian (70% postmenopausal women)	8882	2 weeks—4 years	45–90 mg/day menatetrenone^#^	Yes	–	–	Increased	Decreased ucOC/OC	Non-significant decrease	Increase incidence of adverse effects and adverse drug reactions
Mott et al., 2019 [89]*	Postmenopausal women with or without OP; patients with cirrhosis; patients with chronic glomerular nephritis; healthy population; patient population. East Asian, Caucasian and Iranian populations	11,112	6–48 months	100ug-45 mg VK1 or VK2, 150 mg-3 g Ca/0.5-38ug Vit D. Some studies had corticosteroids or bisphosphonates	–	–	–	Increased. If only low risk of bias studies, no significant difference	–	Decreased in clinical fractures, no difference in vertebral fractures	–
Morato-Martinez et al., 2020 [82]	Postmenopausal Caucasian women with osteopenia	78	24 weeks	Dairy preparation with or without calcium, vitD, VK, vit C, zinc, magnesium, L-leucine and probiotics	–	–	–	Increased	Increased P1NP, decreased CTx	–	
Bartstra et al., 2021 [110]	DM2 and CVD patients	68	6 months	360ug/day VK2	–	–	–	No difference	–	–	No difference on dp-ucMGP
Rønn et al., 2021 [79]	Postmenopausal Caucasian women with osteopenia	142	3 years	375 ug/day MK-7, 38 ug vit D, 800 mg calcium	Yes	–	–	No difference	Increased cOCN; no difference in CTx, P1NP, BSAP, vit D	–	No changes in microstructure of bone
Torbergsen et al., 2019 [81]	Caucasian patients with hip fracture	71	4 months	150 ug VK1, 20 ug vitD and 1000 mg Ca, 250 ug vit A, 10 mg vit E, 1,2 g omega3 fatty acids	Yes	Yes	HPLC	–	Decrease CTx and ucOCN	–	VK1 increased after intervention (not significant when adjusted by baseline differences)
Zhang et al., 2020 [111]	Chinese men and postmenopausal women 50–75 years	311	12 months	50-90ug/day MK-7, 500 mg/day calcium and 10ug/day vit D3	Yes	Yes	ELISA	Decrease in FN in women at 90ug (with and without ca/vitD), but no men	Increased cOCN/ucOCN ratio	–	VK2 increased after 12 months of intervention

*Meta-analysis. ^#^menatetrenone = synthetic form of VK-2, chemically similar to MK-4. *ucOCN* undercarboxylated OCN, *BMD* bone mineral density, *BTM* bone turnover biomarkers, *OP* osteoporosis, *DM2* diabetes mellitus type 2, *CVD* cardiovascular disease, *dp-ucMGP* dephosphorylated-uncarboxylated MGP, *CTX1* Type I collagen cross-linked C-telopeptide, *BSAP* bone specific alkaline phosphatase, *P1NP* total procollagen type I N-terminal propeptide, *cOCN* carboxylated OCN

Bone turnover biomarkers were only analysed in a subset of the available studies (Table 3), with variable results. In some cases, VK increased P1NP and decreased CTx levels [61, 81, 82], but these results were not consistent across the trials [78, 83]. Only undercarboxylated OCN was consistently reduced by VK, which confirms the efficacy of the supplementation.

Quantitative ultrasound sonometry is a widely used tool to assess bone quality. One of the parameters obtained is the speed of sound (SOS) which informs about the bone density. Suzuki et al. [84] found that levels of undercarboxylated OCN negatively correlate with SOS in 49 healthy Japanese females, 84% of which presented VK-1 insufficiency. The mechanism involved is still unknown, but the authors propose that VK could regulate bone quality either as a coenzyme of Gla proteins involved in mineralization [49] or as a ligand for the SXR receptor, to stimulate the expression of TSK, which is involved in collagen assembly and could affect the bone scaffold [56].

Fractures are the typical result of reduced bone quality. Therefore, several studies have investigated if VK is related to fracture risk, but the results do not permit a clear answer yet (as reviewed in [85, 86]). Observational studies, including predominantly healthy and osteoporotic Caucasians, suggest that the serum VK concentration is inversely related to fracture risk in line with previous multicentre studies [87, 88]. In a cross-sectional study of 374 women with postmenopausal osteoporosis, Moore et al. showed a 45% reduction in fracture risk per ug/L higher serum VK-1 concentration [74]. This association remained significant after correcting for age, BMI, vitamin D and lifestyle factors. In addition, VK-1 was also associated with hip geometry and indices of mechanical strength. These results are in line with the NOREPOS study (Norwegian Epidemiologic Osteoporosis Study) [76]. This large epidemiologic study includes 21,774 men and women from four population-based studies aged 65–79 years. After adjustment for age and gender, low serum concentrations of VK-1 and vitamin D were associated with a 50% higher risk for hip fractures.

Intervention studies also have been conducted to investigate the effect of VK supplementation in preventing fractures. The results of these studies are mixed. Whilst some found a significant reduction in fracture risk [61, 77, 89], others did not [80, 90]. A first meta-analysis of osteopenic/osteoporotic patients and controls showed reduction of fracture risk in patients, but not in controls [77]. However, this meta-analysis is limited by the heterogeneity of the studies included. For example, all osteopenic/osteoporotic patients were of East-Asian origin, whereas the healthy controls were mostly Caucasian controls. Furthermore, some studies analysed rather small cohorts, with as few as 20 patients [52]. A later meta-analysis performed by Mott et al. in 2019, included 36 studies with a total of 11,112 participants. In this analysis VK reduced the risk of all clinical fractures in postmenopausal women and osteoporotic patients (OR 0.72, 95% CI 0.55 to 0.95) [89], although after excluding studies with a high risk of bias, the effect was no longer significant (OR 0.76, 95% CI 0.58 to 1.01). However, it should be mentioned that this meta-analysis included a very broad spectrum of healthy individuals and patients with bone, liver and kidney disease from different ethnical groups. The absence of a significant VK effect on fracture risk is further supported by another meta-analysis of Asian cohorts that included 70% of postmenopausal women [90]. A general issue comparing VK supplementation studies is the wide range of doses that were administered, the frequent combination with other micronutrients, anti-osteoporotic drugs and a highly variable study duration.

Additional clues regarding the effect of VK on fracture risk can be drawn from patients that are treated with Warfarin, an inhibitor of GGCX and VKORC1, which causes an inhibition of VK function. Existing studies reported mixed results. However, a meta-analysis including 22 observational studies and one randomized controlled trial, with more than one million participants in total, failed to identify any association between VK antagonists or DOACs and fracture risk, even during long-term treatment. Nevertheless, in selected sub-groups, such as female VK antagonist users (OR 1.11 [95%CI 1.02–1.21]) and patients over 65 years of age (OR 1.07 [95%CI 1.01–1.14]), this meta-analysis found small, but significant associations. Although existing data suggests that the risk of fracture does not seem a major consideration in anticoagulated patients, it should be point out that most existing studies are of observational nature.

Despite the lack of convincing evidence that VK modifies BMD and fracture risk, some recent studies raised the hypothesis that the gut microbiome might be related to fracture risk through its effects on VK metabolism. A small study in 38 postmenopausal women with no conditions or medication affecting their bone metabolism or gut microbiota showed that Bacteroides, which produce MK-10, MK-11 and MK-12, were associated with VK-2 levels and fracture risk [91]. Since no differences in VK intake were found between patients with low and high VK-2 levels, these results suggest a role of gut microbiota in reducing fractures risk by regulating VK-2 levels. The relevance of this observation is further supported by a case report of a 48-year-old Asian man with bisphosphonate-associated fractures. An incomplete atypical femoral fracture on his left leg was treated with 15 mg of VK-2 three times a day. After 4 months, X-rays showed healing of the fracture line, supporting an anabolic effect of VK-2 [92].

### Role of Vitamin K in Sarcopenia

Imbalances of the serum VK status have also been discussed to adversely affect muscle mass and function [93]. Since 2015, only five clinical trials have assessed the role of VK in muscle health (Table 4). Observational studies found that better physical performance is associated with higher plasma concentrations of VK [94]. However, intervention studies have not yet confirmed a causal relationship [95, 96].

**Table 4:** Observational and interventional studies on the effect of vitamin K in muscle function from 2015

Study	Population	N	Duration	Supplementation	ucOCN	ucMGP	Serum VK levels	VK measure method	muscle outcome	other outcomes
Fulton et al., 2016 [95]	Patients with vascular disease < = 70 years	80	6 months	100ug MK-7 daily	–	Yes	–	–	No difference in handgrip strength or SPPB	Decreased ucMGP
Shea et al., 2017 [96]	Older adults	401	3 years	500 ug/day VK1, 600 mg Ca and 400 IU vitD3	Yes	–	–	–	No difference in appendicular lean mass or total body fat mass	Decreased ucOCN
van Ballegooijen et al., 2018 [97]	Adults 55–65 years	633	14 years follow-up	–	–	Yes	–	–	Lower handgrip strength, smaller calf circumference, poorer functional performance in women with high ucMGP	
Beaudart et al., 2019 [112]	Adults > = 65 years	331	2 years follow-up	–	–	–	–	–	sarcopenia associated to less micronutrients (K, Mg, P, Fe, VK)	
Shea et al., 2016 [94]	Older black and white adults with OA	1089	6 years follow-up	–	–	Yes	Yes	reversed-phase HPLC	Better SPPB and 20 m gait speed in higher VK1. Better SPPB and leg strength in lower plasma dp-ucMGP cross-sectionally	

*ucOCN* undercarboxylated OCN, *ucMGP* undercarboxylated MGP

The first study on the role of VK in lower extremity function was carried out in 2016 [94]. In 1,089 elderly participants of the Health, Ageing and Body Composition Study (Health ABC), lower extremity muscle function was assessed by a short physical performance battery (SPPB), gait speed over 20 m, endurance over 400 m and knee extensor strength. Individuals with a plasma VK-1 concentration over or equal to 1 nmol/L (corresponding to 90–120 µg/day dietary intake) showed better lower extremity physical performance, both cross-sectionally and over 5 years of follow-up. In addition, the ucMGP concentration was associated with SPPB cross-sectionally, but not longitudinally. The potential role of ucMGP as a surrogate marker of VK status and physical performance has also been investigated in an ongoing, longitudinal population-based study in Dutch adults (the Longitudinal Aging Study Amsterdam, LASA). In this study, higher ucMGP concentrations (indicating a reduced VK status) were associated with lower handgrip strength and calf circumference. Moreover, in women, high ucMGP levels were also related to a lower physical performance [97].

Whilst some studies support a link between VK and muscle function, others do not. In a 6-months intervention study, treatment of elderly vascular patients with 100 μg/day of VK-2 failed to induce any change in vascular or physical outcome parameters [95]. Similarly, in another randomized controlled supplementation study, 3 years of VK-1 administration did not show any improvement in lean mass or fat mass [96]. However, this treatment reduced undercarboxylated OCN levels between 58 and 61% in both women and men [98].

In order to investigate the role of undercarboxylated OCN in muscle and whether it could be a biomarker for bone-muscle interaction, a randomized controlled multicentre crossover trial has been set up very recently [99]. This study aims to recruit men and women over 60-year-old, measure the undercarboxylated OCN levels in serum before and after acute exercise, and correlate these values with parameters of muscle quality. In a previous study, a low undercarboxylated OCN/OCN ratio was associated with lower muscle strength in young women [100], but so far, this relationship has not been tested in older adults yet.

## Limitations

Many clinical studies have investigated the role of VK in bone and muscle homeostasis with conflicting results, due, in part, to multiple methodological differences between the studies. These include the genetic background and lifestyle of the studied populations, the age of the individuals, the variability of data from self-reported fractures, the presence of bone and muscle diseases, and the changes in other vitamins and triglycerides that could affect VK levels. Furthermore, the power of many studies is compromised by a small sample size and the lack of VK measurement in plasma. Food questionnaires, prothrombin rates, or the carboxylation levels of selected target proteins were often used as surrogates for the participants VK status. However, these markers provide at best a very rough estimate of the supply with VK and do not give any information on individual VK species. Finally yet importantly, many VK treatment studies are hampered by the simultaneous administration of other micronutrients, such as vitamin D and therapeutic drugs that interfere with bone and muscle metabolism.

## Conclusion

Despite a substantial body of literature, our knowledge about VK and its role in bone and muscle is still very limited. This is mainly due to the lack of broadly available analytical methods that allow a reliable quantitation of different VK species in serum and other biological matrices. Furthermore, high quality clinical studies are rare, which limits the value of existing meta-analysis. Preclinical studies suggest some anabolic effects that are mediated by osteoblasts and osteoclasts. However, the underlying mechanisms and the relevance of these effects in humans is still poorly understood. Sufficiently powered high quality studies are needed to further explore the role of VK in the musculoskeletal system. In particular, the VK status should always be assessed by direct measurement with a fully validated and quality controlled method. Supplementation studies should avoid co-treatment with other micronutrients and include a proper control group. A potential additive effect with established pharmaceutical anti-osteoporotic drugs should also be explored. Systematic preclinical studies can help to unravel the molecular effects of VK on bone and muscle cells in vitro and in vivo.

## Data Availability

Availability of data and material is not applicable.
